# ﻿Two new species of *Astrothelium* from Sud Yungas in Bolivia and the first discovery of vegetative propagules in the family Trypetheliaceae (lichen-forming Dothideomycetes, Ascomycota)

**DOI:** 10.3897/mycokeys.95.98986

**Published:** 2023-02-08

**Authors:** Martin Kukwa, Pamela Rodriguez-Flakus, André Aptroot, Adam Flakus

**Affiliations:** 1 Department of Plant Taxonomy and Nature Conservation, Faculty of Biology, University of Gdańsk, Wita Stwosza 59, PL-80-308 Gdańsk, Poland University of Gdańsk Gdańsk Poland; 2 W. Szafer Institute of Botany, Polish Academy of Sciences, Lubicz 46, PL-31-512 Kraków, Poland W. Szafer Institute of Botany, Polish Academy of Sciences Krakow Poland; 3 Laboratório de Botânica / Liquenologia, Instituto de Biociências, Universidade Federal de Mato Grosso do Sul, Avenida Costa e Silva s/n, Bairro Universitário, CEP 79070-900, Campo Grande, Mato Grosso do Sul, Brazil Universidade Federal de Mato Grosso do Sul Mato Grosso do Sul Brazil

**Keywords:** lichens, lichenised fungi, Neotropics, South America, taxonomy

## Abstract

Two new species of *Astrothelium* are described from the Yungas forest in Bolivian Andes. *Astrotheliumchulumanense* is characterised by pseudostromata concolorous with the thallus, perithecia immersed for the most part, with the upper portion elevated above the thallus and covered, except the tops, with orange pigment, apical and fused ostioles, the absence of lichexanthone (but thallus UV+ orange-yellow), clear hamathecium, 8-spored asci and amyloid, large, muriform ascospores with median septa. *Astrotheliumisidiatum* is known only in a sterile state and produces isidia that develop in groups on areoles, but easily break off to reveal a medulla that resembles soralia. Both species, according to the two-locus phylogeny, belong to *Astrothelium* s.str. The production of isidia is reported from the genus *Astrothelium* and the family Trypetheliaceae for the first time.

## ﻿Introduction

Trypetheliaceae Zenker is the core family of the order Trypetheliales Lücking, Aptroot & Sipman and comprises about 500 species and 19 genera (Lücking et al. 2017; Wijayawardene et al. 2022); however, according to Aptroot et al. (2016a), the species diversity is higher. It is predicted that the total number of species is close to 800, with the majority of unrecognised taxa to be found in the Neotropics (Aptroot et al. 2016a). Nevertheless, with about 500 species already known, Trypetheliaceae is one of the three, together with Graphidaceae Dumort. and Pyrenulaceae Rabenh., most speciose families of tropical crustose lichens (Aptroot et al. 2016a; Mendonça et al. 2020).

Species of Trypetheliaceae grow in various, mostly tropical and subtropical ecosystems in Africa, America, Asia and Australia and are important and common elements in the rain and dry forests and savannahs (Aptroot et al. 2016a). Despite that, only recently, the generic concept within the family has been revised and the importance of morphological and chemical characters evaluated using molecular approaches (Lücking et al. 2016a; Hongsanan et al. 2020). This resulted in the recognition of several new species (e.g. Aptroot and Cáceres (2016); Aptroot and Lücking (2016); Aptroot et al. (2016b, 2019, 2022); Flakus et al. (2016); Lücking et al. (2016b); Cáceres and Aptroot (2017); Aptroot and Weerakoon (2018); Hongsanan et al. (2020); Jiang et al. (2022)).

Within Trypetheliaceae, the genus *Astrothelium* Eschw. is the most speciose and comprises about 275 species (Lücking et al. 2017; Wijayawardene et al. 2022). It is characterised by the following features: corticate thallus, ascomata which can be simple, aggregated or forming pseudostromata (often differing in structure and colour) and are immersed to prominent, with apical or eccentric and simple or fused ostioles, hyphal and usually carbonised ascomatal wall (textura intricata), clear or inspersed with oil droplets hamathecium and distoseptate, hyaline, transversely septate or muriform ascospores (Aptroot and Lücking 2016). *Astrothelium*, as presently circumscribed, is paraphyletic and consists of two clades. However, as the relationships between those two clades and the *Aptrootia* Lücking & Sipman and *Architrypethelium* Aptroot, are not fully resolved and supported, the conservative solution was adopted here, with *Aptrootia* and *Architrypethelium* treated as separate genera and all other species retained in the large genus *Astrothelium* (Lücking et al. 2016a).

In Bolivia, 35 species of *Astrothelium* are known so far, of which 12 have been recently described (Flakus et al. 2016). In this paper, we describe two further species from a mountain forest in Sud Yungas in Bolivia, including the peculiar, sterile species with isidia. This is the first time that vegetative lichenised propagules have been reported from the genus and the family Trypetheliaceae. Both species are characterised morphologically, anatomically and chemically. Additionally, a comparison with similar species is provided. The placement of both novel species in *Astrothelium* was corroborated by molecular analyses.

## ﻿Materials and methods

### ﻿Taxon sampling and morphological studies

Our study was based on specimens freshly collected by the authors and deposited at KRAM, LPB and UGDA. Morphology and anatomy were examined using stereo- and compound microscopes (Nikon SMZ 800, Nikon Eclipse 80i DIC; Tokyo, Japan). Sections were prepared manually using a razor blade. Sections and squash mounts were examined in tap water, 10% potassium hydroxide (KOH) (K) or lactophenol cotton blue (LPCB; Sigma-Aldrich, catalogue no. 61335-100ML; St. Louis, Missouri, USA) and amyloid reactions of anatomical structures were tested using Lugol’s solution (I) (Fluka no. 62650-1L-F) or with Lugol’s solution preceded by a 10% KOH treatment (K/I). All photomicrographs showing anatomical characters were made using transmitted differential interference contrast (DIC) microscopy. All measurements were made in distilled water. Lichen substances were investigated by thin-layer chromatography (TLC) following the methods by Culberson and Kristinsson (1970) and Orange et al. (2001).

### ﻿DNA extraction, PCR amplification and DNA sequencing

Freshly collected hymenia or thallus fragments were removed from the specimens and carefully cleaned in double-distilled water (ddH_2_O) on a microscope slide under sterile conditions to remove any visible impurities using ultra-thin tweezers and a razor blade. Genomic DNA was extracted from a few ascomata or thallus pieces using the QIAamp DNA Investigator Kit (Qiagen, Hilden, Germany) following the manufacturer’s instructions. We amplified both the mtDNA small subunit DNA (mtSSU) using primers pair mrSSU1 and mrSSU3R (Zoller et al. 1999) and nuc rDNA large subunit (nuLSU) with primers ITS1F, LROR, LR3 and LR5 (Vilgalys and Hester 1990; Rehner and Samuels 1994). Polymerase chain reactions (PCR) were performed in a volume of 25 μl comprising 1 μl of DNA template, 0.2 μl of AmpliTaq 360 DNA polymerase (Applied Biosystems, California, USA), 2.5 μl of 10× AmpliTaq 360 PCR Buffer, 2.5 μl 25mM MgCl_2_, 1 μl of each primer (10 μM), 2 μl GeneAmp dNTPs (10 mM; Applied Biosystems, California, USA), 0.2 μl bovine serum albumin (BSA; New England Biolabs, Massachusetts, USA) and sterile distilled water was added to attain the final volume. PCR amplifications were performed using the thermocycling conditions of Rodriguez-Flakus and Printzen (2014). PCR products were visualised by running 3 μl of the PCR product on 1% agarose gels. PCR amplicons were purified using the ExoSAP method (EURx, Gdańsk, Poland) and sequenced by Macrogen (Amsterdam, the Netherlands). The newly-generated mtSSU and nuLSU sequences were checked, assembled and edited manually using Geneious Pro 8.0. (Biomatters, Auckland, New Zealand) and deposited in GenBank.

### ﻿Phylogenetic analyses and taxon selection

All sequences generated were checked by BLAST (Altschul et al. 1990) to verify potential contaminations by an unrelated fungus. BLAST searches of both mtSSU and nuLSU rDNA sequences from both species revealed the highest similarity with members of *Astrothelium* (Trypetheliaceae, Dothideomycetes). Therefore, we aligned our sequences with the available sequences of the members of *Astrothelium* (Lücking et al. 2016a) (Table 1). Alignments were generated for each region using MAFFT (Katoh et al. 2005) as implemented on the GUIDANCE2 Web server (Penn et al. 2010). GUIDANCE2 assigns a confidence score to each ambiguous nucleotide site in the alignment and later removes regions of uncertain columns. We used the default cut-off score of 0.93 in all single gene alignments. The following analyses were performed in the CIPRES Scientific Gateway (Miller et al. 2010). Maximum Likelihood (ML) analyses were carried out in each single-locus alignment using IQ-TREE version 2.1.2 (Nguyen et al. 2015; Chernomor et al. 2016) to detect potential conflicts. We performed 1000 ultrafast bootstrap replicates to estimate branch support amongst the two loci which later were concatenated to a single alignment. The concatenated dataset was used as an input file for analysing the ML in our studies. In which, we performed 5000 replicates under the best-fitting substitution model determined by the ModelFinder Plus (MFP) as implemented in IQ-TREE (Kalyaanamoorthy et al. 2017). The selected model was GTR+F+I+G2 according to AICc in our partitioned per each locus dataset (gene partitioned -s and -m + MFP + MERGE). Bayesian Inference (BI) of the phylogenetic relationships was calculated using the Markov Chain Monte Carlo (MCMC) approach as implemented in MrBayes 3.2.6 on XSEDE (Ronquist et al. 2012) using the partitions and substitution models obtained. Two independent parallel runs were started each with four incrementally heated (0.15) chains. This MCMC was allowed to run for 40 million generations, sampling every 1000^th^ tree and discarding the first 50% of the sampled tree as a burn-in factor. The resulting ML and BI phylogenetic trees were visualised in TreeView (Page 1996). The tree was rooted by using *Architrypethelium* and *Aptrootia* species as the outgroups.

**Table 1.** Voucher data and GenBank accession numbers for the sequences included in this study. Newly-generated sequences are shown in bold.

Taxon	Origin	Collector	Voucher	Herbarium	Isolate	GenBank accession numbers	
						mtSSU	nuLSU
*Aptrootiaelatior*	New Zealand	Knight	O61815	OTA	MPN560B	KM453821	KM453754
*Aptrootiarobusta*	Australia	Lumbsch	20012	F	MPN235B	KM453822	KM453755
*Aptrootiaterricola*	Costa Rica	Lücking	17211	F	DNA1501	DQ328995	KM453756
*Architrypetheliumlauropaluanum*	Peru	Nelsen	Cit1P	F	MPN48	KX215566	KX215605
*Architrypetheliumnitens*	Panama	Lücking	27038	F	MPN257	KM453823	KM453757
*Architrypetheliumuberinum*	Brazil	Nelsen	s.n.	F	MPN489	–	KM453758
*Astrotheliumaenascens 1*	Thailand	Luangsuphabool	27887	RAMK	HRK93	LC128018	LC127403
*Astrotheliumaenascens 2*	Thailand	Luangsuphabool	27888	RAMK	HRK98	LC128019	LC127404
*Astrotheliumaeneum*	Panama	Lücking	27056	F	MPN302	–	KX215606
*Astrotheliumbicolor*	USA	Nelsen	4002a	F	MPN139	GU327706	GU327728
*Astrotheliumcarassense*	Brazil	Lücking	31004	F	MPN438	KM453849	KM453784
*Astrotheliumcecidiogenum*	Costa Rica	Lücking	s.n.	F	N/A	DQ328991	–
***Astrotheliumchulumanense***	Bolivia	Flakus	29985	KRAM	14-31	**OQ275191**	**OQ281430**
*Astrotheliumcinereorosellum 2*	Philippines	RivasPlata	2106	F	MPN199C	–	KX215610
*Astrotheliumcinereorosellum 1*	Philippines	RivasPlata	2110	F	MPN191	KM453873	KM453809
*Astrotheliumcinnamomeum*	Costa Rica	Lücking	15322b	DUKE	AFTOL110	AY584632	AY584652
*Astrotheliumcrassum*	Peru	Nelsen	s.n.	F	MPN98	GU327685	GU327710
Astrotheliumaff.crassum	Brazil	Cáceres	6011	F	MPN335	KM453827	KM453761
*Astrotheliumcroceum*	Peru	Nelsen	211D	F	MPN55	KX215567	KX215611
*Astrotheliumdegenerans 1*	Costa Rica	Lücking	17502b	CR	DNA1496	DQ328987	–
*Astrotheliumdegenerans 2*	Panama	Lücking	27109	F	MPN267	KM453835	KM453770
*Astrotheliumdiplocarpum 2*	Nicaragua	Lücking	28529	F	MPN210	KM453846	KM453781
*Astrotheliumdiplocarpum 1*	USA	Nelsen	s.n.	F	MPN134	KX215568	–
*Astrotheliumendochryseum*	Brazil	Lücking	31088	F	MPN436	KM453837	KM453772
*Astrotheliumerubescens*	Peru	Nelsen	AnaG	F	MPN96	KX215569	KX215614
*Astrotheliumeuthelium 1*	Thailand	Lücking	24075	F	MPN226	–	KX215615
*Astrotheliumeuthelium 2*	Philippines	RivasPlata	1194B	F	MPN22B	–	KX215616
*Astrotheliumflavocoronatum 1*	Thailand	Luangsuphabool	27890	RAMK	KY859	LC128014	LC127398
*Astrotheliumflavocoronatum 2*	Thailand	Luangsuphabool	27889	RAMK	TSL63	AB759874	LC127397
*Astrotheliumfloridanum 1*	USA	Nelsen	4008	F	MPN132	GU327705	GU327727
*Astrotheliumfloridanum 2*	Panama	Lücking	27131a	F	MPN304	KM453876	KM453811
*Astrotheliumgigantosporum*	Panama	Lücking	33037	F	MPN590	KM453851	KM453786
*Astrotheliumgrossum 2*	Panama	Lücking	27045	F	MPN259	KM453834	KM453769
*Astrotheliumgrossum 1*	Peru	Nelsen	4000a	F	MPN47	GU327689	GU327713
*Astrotheliuminspersoaeneum*	Peru	Nelsen	Cit1K	F	MPN45	KX215571	–
***Astrotheliumisidiatum***	Bolivia	Flakus	30000	KRAM	14-8	**OQ275190**	**OQ281431**
*Astrotheliumkunzei 1*	Salvador	Lücking	28120	F	MPN201B	–	KX215624
*Astrotheliumkunzei 2*	Salvador	Lücking	28137	F	MPN203B	–	KX215625
*Astrotheliumlaevigatum*	Brazil	Lücking	31010	F	MPN430	KX215572	–
*Astrotheliumlaevithallinum*	Brazil	Lücking	31061	F	MPN442	KM453836	KM453771
*Astrotheliumleucoconicum*	Peru	Nelsen	4000c	F	MPN42	KM453830	KM453764
*Astrotheliumleucosessile 1*	Panama	Lücking	27059	F	MPN258	KM453828	KM453762
*Astrotheliumleucosessile 2*	Brazil	Cáceres	11201	F	MPN713	KM453869	KM453805
*Astrotheliummacrocarpum 1*	Panama	Lücking	27077	F	MPN260	KM453829	KM453763
*Astrotheliummacrocarpum 2*	Thailand	n/a	27892	RAMK	UBN37	LC128015	LC127400
*Astrotheliummacrocarpum 3*	Thailand	n/a	27894	RAMK	UBN43	LC128016	LC127399
*Astrotheliummacrostiolatum*	Thailand	Luangsuphabool	27895	RAMK	PHL84	LC128022	LC127407
*Astrotheliummegaspermum 2*	Gabon	Ertz	9725	BR	AFTOL2094	GU561847	FJ267702
*Astrotheliummegaspermum 3*	USA	Nelsen	s.n.	F	MPN138	KX215574	KX215632
*Astrotheliummegaspermum 1*	Thailand	Nelsen	s.n.	F	MPN32B	KX215576	–
*Astrotheliummeristosporum 2*	Philippines	RivasPlata	2128	F	MPN198	–	KX215634
*Astrotheliummeristosporum 1*	Philippines	RivasPlata	2108	F	MPN189	KM453850	KM453785
*Astrotheliumneglectum 1*	Thailand	Luangsuphabool	27898	RAMK	TAK8	LC128025	LC127410
*Astrotheliumneglectum 2*	Thailand	Luangsuphabool	27896	RAMK	TAK12	LC128026	LC127411
*Astrotheliumneglectum 3*	Thailand	Luangsuphabool	27897	RAMK	TAK17	LC128027	LC127412
*Astrotheliumneogalbineum 1*	Brazil	Cáceres	11100	F	MPN711	KM453877	KM453812
*Astrotheliumneogalbineum 2*	Peru	Nelsen	Cit1T	F	MPN51	KX215577	KX215635
*Astrotheliumneoinspersum 2*	Peru	Nelsen	AnaJ	F	MPN61C	–	KX215636
*Astrotheliumneoinspersum 1*	Peru	Nelsen	s.n.	F	MPN62	KM453866	KM453802
*Astrotheliumneovariolosum 1*	Thailand	Luangsuphabool	27899	RAMK	KY777	LC128023	LC127408
*Astrotheliumneovariolosum 2*	Thailand	Luangsuphabool	27900	RAMK	KY848	LC128024	LC127409
*Astrotheliumnicaraguense 1*	Nicaragua	Lücking	28503	F	MPN205	–	KX215637
*Astrotheliumnicaraguense 2*	Nicaragua	Lücking	28551	F	MPN213	–	KX215639
*Astrotheliumnitidiusculum 2*	Fiji	Lumbsch	20547i	F	MPN768	–	KX215640
*Astrotheliumnitidiusculum 1*	Brazil	Cáceres	11297	F	MPN704	KM453868	KM453804
*Astrotheliumnorisianum*	Peru	Nelsen	4000d	F	MPN52C	KM453848	KM453783
Astrotheliumaff.norisianum	Peru	Nelsen	Cit1B	F	MPN23B	KX215578	KX215607
Astrotheliumaff.obscurum	Philippines	RivasPlata	2175	F	MPN194	–	KX215608
*Astrotheliumobtectum*	Brazil	Lücking	31242	F	MPN422	KM453832	KM453767
*Astrotheliumperspersum*	Gabon	Ertz	9716	BR	AFTOL2099	GU561848	FJ267701
*Astrotheliumphlyctaena 1*	USA	Nelsen	4167	F	MPN373	–	KX215641
*Astrotheliumphlyctaena 2*	USA	Nelsen	4149	F	MPN386	–	KX215644
*Astrotheliumpulcherrimum*	Panama	Lücking	27046	F	MPN313	KM453879	KM453814
*Astrotheliumpupula*	Colombia	Lücking	26305	F	MPN224	KM453880	KM453815
*Astrotheliumpurpurascens*	Peru	Nelsen	s.n.	F	MPN53C	KM453847	KM453782
*Astrotheliumrobustum 1*	Costa Rica	Mercado	586	F	MPN754	KM453826	KM453760
*Astrotheliumrobustum 2*	Nicaragua	Lücking	28519	F	MPN209	–	KX215645
*Astrotheliumrobustum 3*	Nicaragua	Lücking	28547	F	MPN212	–	KX215646
*Astrotheliumrufescens 1*	Brazil	Nelsen	B1	F	MPN143	–	KX215650
*Astrotheliumrufescens 2*	Argentina	Lücking	30511	CTES	MPN346	–	KX215652
*Astrotheliumsanguinarium 1*	Brazil	Cañez	3133	CGMS	MPN765	KM453853	KM453788
*Astrotheliumsanguinarium 2*	Brazil	Cañez	3135	CGMS	MPN766	KX215579	KX215653
*Astrotheliumsanguinarium 3*	Brazil	Cañez	3137a	CGMS	MPN767	KX215580	KX215654
*Astrotheliumscoria*	Panama	Lücking	27181	F	MPN310	–	KX215655
*Astrotheliumscorizum*	Brazil	Lücking	29814	F	MPN336	KM453872	KM453808
Astrotheliumaff.sepultum 2	Costa Rica	Lücking	21027	F	MPN229	–	KX215609
Astrotheliumaff.sepultum 1	Peru	Nelsen	4001a	F	MPN63C	GU327690	GU327714
*Astrotheliumsiamense 1*	Thailand	Luangsuphabool	27901	RAMK	KRB105	LC128020	LC127405
*Astrotheliumsiamense 2*	Thailand	Luangsuphabool	27902	RAMK	KRB139	LC128021	LC127406
*Astrotheliumsubcatervarium*	Peru	Nelsen	4009a	F	MPN97	GU327707	GU327729
*Astrotheliumsubendochryseum*	Salvador	Lücking	28121	F	MPN202B	–	KX215659
*Astrotheliumsubinterjectum*	Brazil	Nelsen	B15	F	MPN157	KX215583	KX215660
*Astrotheliumsubscoria 1*	Nicaragua	Lücking	28640	F	MPN217	KM453878	KM453813
*Astrotheliumsubscoria 2*	Bolivia	Lücking	29010	F	MPN325	KX215584	KX215661
*Astrotheliumtuberculosum*	Costa Rica	Lücking	16306a	F	DNA1504	DQ329008	–
*Astrotheliumvariolosum 1*	Peru	Nelsen	s.n.	F	MPN43	KM453833	KM453768
*Astrotheliumvariolosum 2*	Peru	Nelsen	Cit1F	F	MPN41	KX215585	KX215662

## ﻿Results and discussion

**Figure 1.**
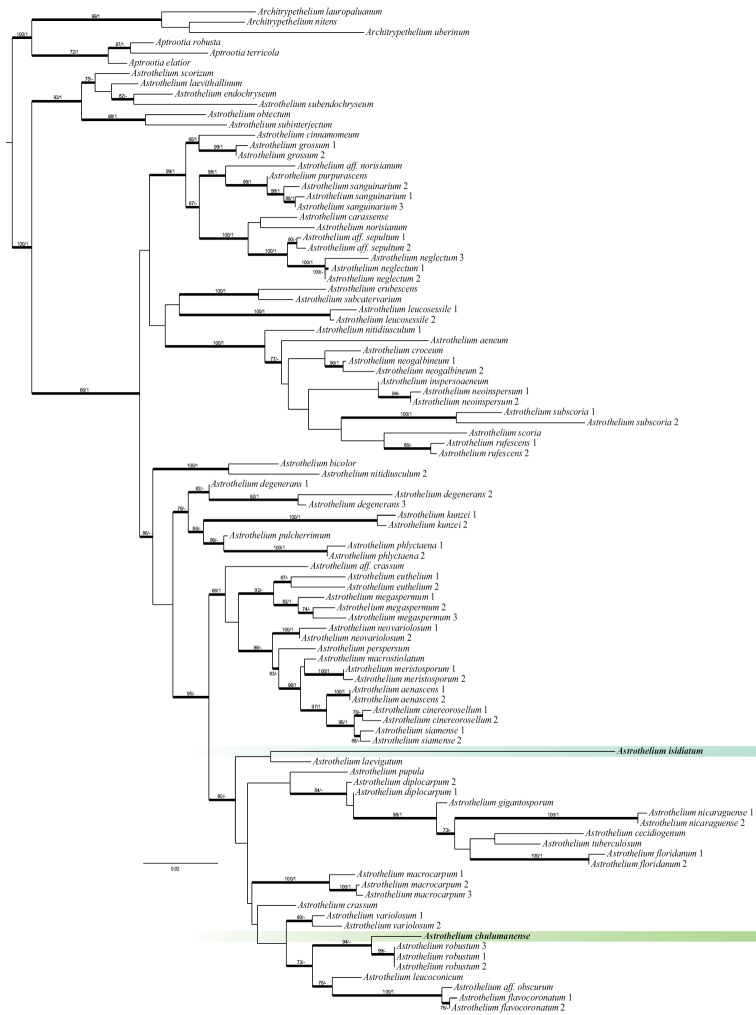
Phylogenetic placement of the two new species of *Astrothelium* within Trypetheliaceae inferred from ML analyses of combined mtSSU and nuLSU rDNA dataset. *Aptrootia* and *Architrypethelium* species were used as the outgroups. Bold branches represent either bootstrap values ≥ 70 and/or Bayesian posterior probabilities ≥ 0.95.

Two new sequences of each marker (mtSSU and nuLSU) from two new species of *Astrothelium* were generated for this study (Table 1). The final DNA alignment consisted of sequences obtained from 98 specimens and two markers with a total of 1128 characters, 487 distinct patterns, 288 parsimony-informative, 102 singleton sites and 738 constant sites. The ML phylogenetic tree is presented in Fig. 1.

The phylogenetic reconstruction shows that all *Astrothelium* species form a well-supported clade divided into two subclades, of which the smaller and well-supported (six species) refers to the clade labelled as *Astothelium* s.lat. by Lücking et al. (2016a) and the larger one refers to *Astrothelium* s.str., but is poorly supported (Fig. 1). Our results differ from those received by Lücking et al. (2016a) as all species of *Astrothelium*, although still divided into two groups, form one clade, with *Aptrootia* and *Architrypethelium* forming the sister clade. However, our analyses were restricted only to *Astrothelium* and two related genera, *Aptrootia* and *Architrypethelium*.

*Astrotheliumchulumanense* and *A.isidiatum* are placed in the larger clade defined by Lücking et al. (2016a) as *Astrothelium* s.str. *Astrotheliumchulumanense* forms a strongly-supported clade together with *A.robustum* Müll. Arg.; however, the relationship of this two-species clade with other species within *Astrothelium* s.str. is not well resolved (Fig. 1). *Astrotheliumisidiatum* is grouped with *A.laevigatum* Müll. Arg., but the support is weak (Fig. 1). In addition, the relationships of this two-species clade within *Astrothelium* s.str. are not supported.

The most surprising finding is the presence of isidia in one of the new species, *Astrotheliumisidiatum*. This is the first case when vegetative lichenised diaspores are reported in Trypetheliaceae. Moreover, the new species is sterile and lichen taxa being sterile, but reproducing by isidia or other similar propagules consisting of mycobiont and photobiont, are known in several other groups of lichenised fungi. In extreme cases even entire lineages evolved into permanently asexually reproducing genera, like *Botryolepraria* Canals et al., *Lepraria* Ach. and others (Canals et al. 1997; Ekman and Tønsberg 2002; Kukwa and Pérez-Ortega 2010; Hodkinson and Lendemer 2013; Lendemer and Hodkinson 2013; Guzow-Krzemińska et al. 2019). In some genera, sterile taxa producing vegetative diaspores prevail, like in *Herpothallon* Tobler (Aptroot et al. 2009), but in others, they are rarer, for example, in *Ochrolechia* A. Massal. (Kukwa 2011). It seems that, in groups of perithecioid lichens, they are much rarer than in apothecioid lichens (e.g. Diederich and Ertz (2020); Orange and Chhetri (2022)). *Astrotheliumisidiatum* is the first species of the Trypetheliaceae, as mentioned above, reproducing by lichenised propagules. However, it is highly possible that more such taxa can be discovered in poorly-explored areas, like Bolivian and other South American ecosystems, but such sterile lichens cause difficulties in placing them properly in higher taxa without molecular approaches; therefore, they can be easily omitted in taxonomic revisions. Additionally, they may have more inconspicuous thalli compared to fertile species (thallus areoles of *A.isidiatum* were found dispersed amongst other lichens) and can be easily overlooked.

The two new species of *Astrothelium*, as well as some of these recently described taxa within Trypetheliaceae from Bolivia by Flakus et al. (2016), may be potentially endemic to some areas in this country. With tens of thousands of samples collected by our team across all major ecosystems in Bolivia over almost 20 years, single or only very few records of each new species have been found (Flakus et al. 2016), which may suggest their restricted distribution. This situation can be similar to the genus *Sticta* (Schreb.) Ach. in which several species are confined only to some regions (Moncada et al. 2014, 2018, 2020; Dal Forno et al. 2018; Simon et al. 2018; Mercado-Díaz et al. 2020; Ossowska et al. 2022).

### ﻿Taxonomy

#### ﻿ Astrothelium chulumanense

Taxon classificationFungiTrypethelialesTrypetheliaceae

Flakus, Kukwa & Aptroot sp. nov.

452E1F6A-6B9B-54EC-9518-668F45C88546

MycoBank No: 847215

Fig. 2

##### Diagnosis.

Characterised by pseudostromata not differing in colour from the thallus, perithecia immersed for the most part in thallus, with the upper part elevated above the thallus and covered, except the tops, with orange pigment, apical and fused ostioles, the absence of lichexanthone, clear hamathecium, 8-spored asci and amyloid, large (125–167 × 27–35 μm), muriform ascospores with a thickened median septum.

**Figure 2.**
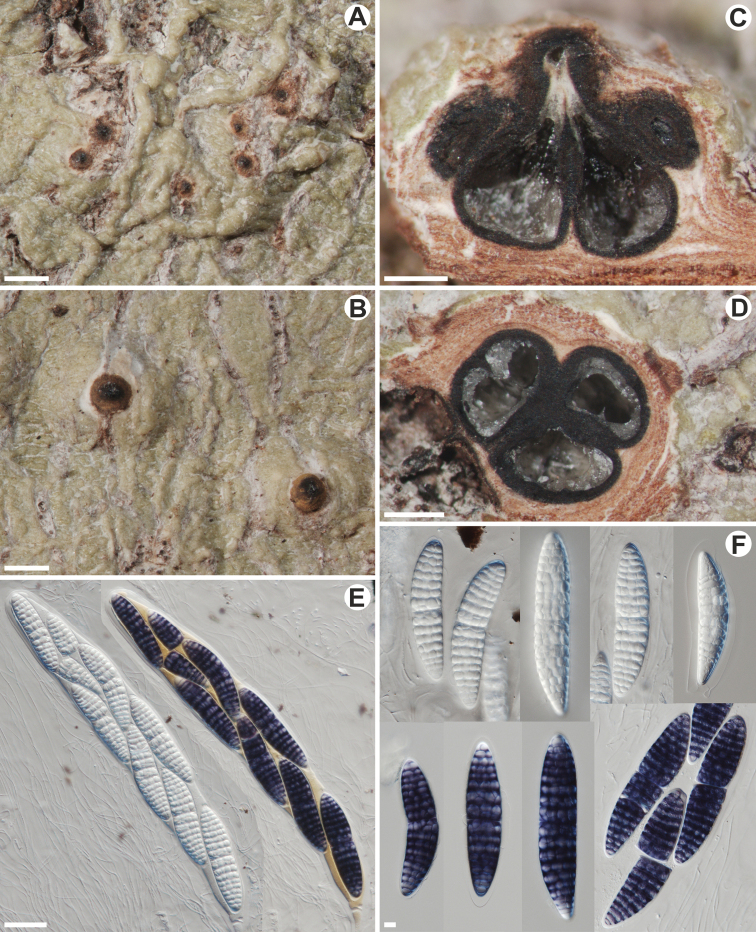
*Astrotheliumchulumanense* (holotype) **A, B** thallus and ascomata **C** vertical cross section through pseudostromata **D** horizontal cross section through pseudostromata **E** asci (violet ascospores in Lugol’s solution) **F** ascospores (violet in Lugol’s solution). Scale bars: 1000 μm (**A, B**); 500 μm (**C, D**); 50 μm (**E**); 10 μm (**F**).

##### Type.

Bolivia. Dept. La Paz; Prov. Sud Yungas, Pataloa, near estación biológica Santiago de Chirca, near Chulumani, 16°23'57.16"S, 67°34'33.96"W, elev. 2271 m, Yungas montane forest, corticolous, 22 Jan 2020, A. Flakus 29985 & P. Rodriguez-Flakus (holotype KRAM-L 73244, isotypes LPB, UGDA).

##### Description.

Thallus corticate, with corticiform layer 10–20 μm thick, uneven, folded to bumpy, somewhat shiny, continuous, ca. 0.1mm thick, greenish, surrounded by a dark prothallus, not inducing swellings of the host bark, covering areas ≤ 8 cm diam. Pseudostromata with a surface similar to the thallus, distinctly raised above the thallus, hemispherical to wart-shaped, ca. 1.5–3 mm in diam. and 0.5–1.5 mm high, the same colour like thallus with black to orange-black apical spot, inside containing bark tissue. Ascomata perithecia, pyriform to hemispherical, aggregated, 0.6–1 mm diam., emerging from beneath the upper periderm layers of the bark and surrounded by bark tissues in outside part, immersed in most parts in regular in outline pseudostromata, upper part elevated above the thallus and covered, except the tops, with orange pigment. Ostioles apical, centrally fused to form a shared channel leading to various chambers. Wall fully carbonised, not differentiated into excipulum and involucrellum, thicker, ≤ ca. 100 μm wide in the upper part and thinner, up to ca. 20 μm wide, near the base. Ostioles apical, fused, black. Hamathecium clear, composed of thin and anastomosing paraphysoids, 1.5–2.5 μm wide. Asci 8-spored, 350–470 × 56–60 µm. Ascospores distoseptate, hyaline, I+ violet, densely muriform, with a gelatinous layer in younger stages, with a distinct thickened median septum, sometimes breaking into two parts in the septa, narrowly ellipsoid, 125–167 × 27–35 μm, ends rounded, lumina diamond-shaped.

##### Chemistry.

Thallus surface UV+ orange-yellow, K–, C–, KC–, thallus medulla K–; pseudostromata surface UV+ orange-yellow, K–, inner part of pseudostromata K–, visible part of perithecia K+ red. Trace of unidentified substance detected in the thallus by thin layer chromatography; pigment on the top of perithecia.

##### Etymology.

The species is named after its locus classicus located near Chulumani town in Bolivia.

##### Distribution and habitat.

So far, the species is known only from the type locality in Yungas forest in Bolivia.

##### Notes.

*Astrotheliumchulumanense* can be distinguished by pseudostromata not differing in colour from the thallus, the orange-yellow reaction in UV (perhaps due to the presence of an unknown substance), the absence of lichexanthone, perithecia immersed for the most part in the thallus, but with upper part elevated above the thallus and covered, except the tops, with orange pigment, apical and fused ostioles, clear hamathecium, 8-spored asci and amyloid, large, muriform ascospores with median septa. The new species is phylogenetically related and externally similar to *A.robustum*. Both species have also ascomata with fused ostioles; however, ascospores in *A.robustum* are (3–)5–7(–9)-septate and I negative. Furthermore, the species does not produce secondary metabolites (Aptroot and Lücking 2016; Aptroot 2021).

Only four *Astrothelium* species have clear hamathecium, 8-spored asci and large, muriform ascospores, which react I+ violet. *Astrotheliumamylosporum* Flakus & Aptroot has pseudostromata not covered by thallus and lacks pigments, whereas *A.palaeoexostemmatis* Sipman & Aptroot lacks pigments, has smaller ascospores (85–100 × 20–24 μm) and ascomata are almost completely covered by the thallus and do not form distinct pseudostromata. *Astrotheliumsanguinarium* (Malme) Aptroot & Lücking differs in the shape of pseudostromata, the pigment is red (isohypocrellin), reacts K+ yellow-green and is present internally within pseudostromata. *Astrotheliumsanguineoxanthum* Aptroot has smaller (up to 86 μm long) ascospores, whitish pseudostromata and produces lichexanthone and isohypocrellin (internal in pseudostromata) (Aptroot and Lücking 2016; Aptroot et al. 2016b, 2019; Flakus et al. 2016; Aptroot 2021).

Several other species of the genus have pseudostromata or aggregated ascomata often with fused ostioles, clear hymenium, large (at least some over 80 μm long) and muriform, but I negative ascospores and 8-spored asci. They differ significantly in other characters (for the key to all species, see Aptroot (2021)). In *A.alboverrucum* (Makhija & Patw.) Aptroot & Lücking, ascomata are solitary to diffusely pseudostromatic, prominent, with whitish surrounding the black ostiolar area (Aptroot and Lücking 2016). *Astrotheliumcarassense* Lücking, M. P. Nelsen & Marcelli differs in perithecia completely immersed in pseudostromata, which are covered with orange pigment (Lücking et al. 2016b). *Astrotheliumchapadense* (Malme) Aptroot & Lücking differs in dark brown pseudostromata, up to 100 μm long ascospores and the lack of secondary metabolites (Aptroot and Lücking 2016). *Astrotheliumconfluens* (Müll. Arg.) Aptroot & Lücking has ascomata completely covered by the thallus and ascospores measuring ca. 130 × 20 μm (Aptroot and Lücking 2016). *Astrotheliumdefossum* (Müll. Arg.) Aptroot & Lücking has joined ascomata, which are dispersed to confluent or diffusely pseudostromatic with lichexanthone on the surface (Aptroot and Lücking 2016). *Astrotheliumelixii* Flakus & Aptroot develops white pruinose pseudostromata and produces lichexanthone and isohypocrellin (internal in pseudostromata) (Flakus et al. 2016). *Astrotheliumflavoduplex* Aptroot & M. Cáceres differs from the new species by the presence of lichexanthone, oval to irregular or reticulate in outline pseudostromata, which are yellow to brownish and contain up to 50 ascomata with no fused ostioles (Aptroot and Cáceres 2016). *Astrotheliumflavomurisporum* Aptroot & M. Cáceres has aggregated ascomata (but without pseudostroma) covered with the thallus, lumina of ascospores with yellow oil and lacks secondary metabolites (Aptroot and Cáceres 2016). *Astrotheliummegeustomum* Aptroot & Fraga Jr produces ascomata mostly immersed in the bark tissue below pseudostromata, up to 125 μm long ascospores and lichexanthone around ostiolar region (Aptroot et al. 2016b). *Astrotheliummesoduplex* Aptroot & M. Cáceres has ascomata immersed in superficially yellow to orange, pale yellow inside pseudostromata and shorter, up to 100 μm long ascospores (Aptroot and Cáceres 2016). *Astrotheliumoctosporoides* Aptroot & Lücking differs in solitary or a few grouped ascomata covered by the thallus and the lack of secondary metabolites (Aptroot and Lücking 2016). *Astrotheliumpurpurascens* (Müll. Arg.) Aptroot & Lücking develops ascomata with fused ostioles covered with the thallus, produces isohypocrellin and has mostly shorter ascospores (100–130 μm) (Aptroot and Lücking 2016). *Astrotheliumvariabile* Flakus & Aptroot has aggregated ascomata in well-delimited and white pseudostromata, not fused ostioles, lacks pigments and produces lichexanthone (Flakus et al. 2016). *Astrotheliumxanthosuperbum* Aptroot & M. Cáceres differs in black, raised above the thallus pseudostromata, which are usually in lines, the lack of pigments and the production of lichexanthone (Aptroot and Cáceres 2016).

#### ﻿ Astrothelium isidiatum

Taxon classificationFungiTrypethelialesTrypetheliaceae

Kukwa, Flakus & Rodr. Flakus sp. nov.

AD26A0AB-7FDF-5285-9171-C39024CE4226

MycoBank No: 847216

Fig. 3

##### Diagnosis.

The new species differs from all known species of the genus by developing groups of isidia on the surface of areoles, which break off to reveal a medulla that resembles soralia.

##### Type.

Bolivia. Dept. La Paz; Prov. Sud Yungas, near Reserva Ecológica de Apa Apa, Sanani near Chulumani, 16°20'39.70"S, 67°29'54.32"W, elev. 2423 m, Yungas montane forest, corticolous, 23 Jan 2020, A. Flakus 30000 & P. Rodriguez-Flakus (KRAM-L 73245 holotype; LPB, UGDA isotypes).

##### Description.

**Figure 3.**
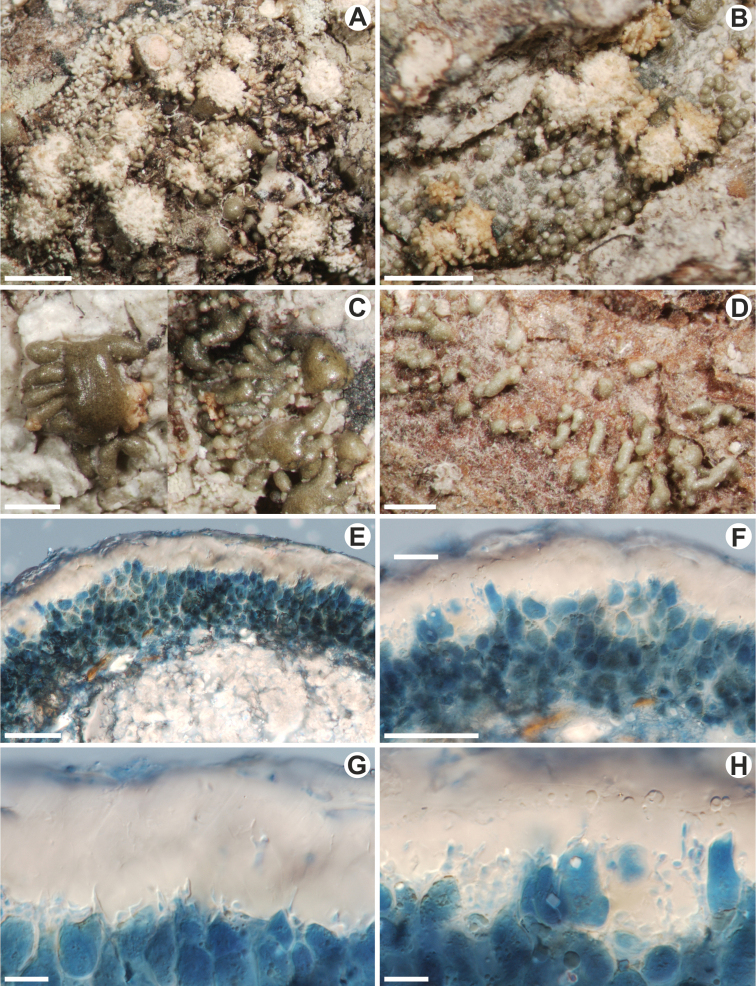
*Astrotheliumisidiatum* (type collection) **A–D** thallus morphology **A, B** isidia developing in groups on areoles which are partly shed exposing the medulla of the areoles **C** isidia-like outgrows developing on lateral parts of areoles **D** isidia-like outgrowths developing directly from the endosubstratal parts of the thallus **E, F** a vertical cross-section through thallus with crystals present in the medulla (**E**) (in LPCB) **G, H** vertical cross-section through cortical layer (in LPCB). Scale bars: 1000 μm (**A, B**); 500 μm (**C, D**); 50 μm (**E, F**); 10 μm (**G, H**).

Thallus endosubstratal to episubstratal and then grey-green, shiny, folded in non-areolate parts, with areoles, isidiate. Areoles tuberculate, sometimes with cylindrical outgrowth developing at the lateral parts of areoles (Fig. 3C), constricted at the base (especially when young) or not, rounded to elongate and up to 1.2 mm wide. Isidia mostly cylindrical, globose when young, simple, rarely branched, constricted at the base or not, developing on areoles, up to 0.5 mm long and 0.2 mm wide, often shed from areoles and then exposing the yellow medulla of areoles, which then resemble soralia; sometimes elongated isidia-like outgrowth developing directly from the endosubstratal thallus present (Fig. 3D). Cortex up to 30–50 µm in width, of two layers, lower part prosoplectenchymatous and visible mostly in young areoles and upper part gelatinous. Photobiont layer up to 35 µm wide. Medulla whitish (only in young areoles) to yellow, densely filled with rhomboid or irregular crystals (crystals not dissolving in K), crystals 4–35 × 3–12 µm. The upper layer of areoles with shed isidia pseudoparenchymatous. Ascomata and pycnidia unknown.

##### Chemistry.

Thallus surface UV–, K–, C–, KC–; medulla with yellow pigment, K+ yellow going into solution, C+ yellow-orange; upper parts of areoles with shed isidia with patches of orange pigment reacting K+ purple. Unidentified substances (probably some of them are anthraquinones) in trace to minor amounts detected by thin layer chromatography.

##### Etymology.

The name refers to the production of isidia, which are unique in the genus.

##### Distribution and habitat.

So far, the species is known only from the type locality in the Yungas forest in Bolivia.

##### Notes.

This is a very characteristic species with areoles filled with crystals, cylindrical isidia developing on the areoles and usually yellow thallus medulla. The ascomata were not found in the studied material. It differs from all species of *Astrothelium* and Trypetheliaceae in the presence of isidia.

Some species of Trypetheliaceae, for example, *Architrypetheliumlauropaluanum* Lücking, M. P. Nelsen & Marcelli, *Astrotheliumkomposchii* Aptroot or *A.puiggarii* (Müll. Arg.) Aptroot & Lücking (Aptroot and Lücking 2016; Aptroot et al. 2016c; Lücking et al. 2016b), develop thalli with areoles resembling isidia which somehow are similar to these of *A.isidiatum* (Fig. 3C, D). However, *A.isidiatum* differs by developing cylindrical and often constricted at the base isidia which are covering the entire areoles (Fig. 3A, B). The isidia are easily broken and shed from areoles revealing the medulla of areoles that then resemble soralia.

We are not aware of any other similar species in other groups, which remind us of the unique taxon described here.

## Supplementary Material

XML Treatment for Astrothelium chulumanense

XML Treatment for Astrothelium isidiatum
